# Extracellular vesicles derived from TNF-α-preconditioned mesenchymal stem cells mitigate inflammatory retinal injury

**DOI:** 10.20517/evcna.2025.159

**Published:** 2026-03-17

**Authors:** Zhuxin Jia, Fuxiao Luan, Jingyi Shi, Yong Tao, Ying Tian

**Affiliations:** ^1^Department of Ophthalmology, Beijing Chaoyang Hospital, Capital Medical University, Beijing 100020, China.; ^2^National Engineering Research Center for Ophthalmology, Beijing 102600, China.; ^3^Engineering Research Center of Ophthalmic Equipment and Materials, Ministry of Education, Beijing 100176, China.; ^4^Chinese Institutes for Medical Research, Beijing 100071, China.

**Keywords:** TNF-α, mesenchymal stem cell, small extracellular vesicles, sEV-derived miRNAs, inflammatory retinal injury

## Abstract

**Aim:** To evaluate the therapeutic efficacy and mechanisms of tumor necrosis factor-alpha (TNF-α) preconditioned mesenchymal stem cells (MSCs)-derived small extracellular vesicles (hereafter abbreviated as T-sEV), along with the control small extracellular vesicles (sEV, representing vesicles from naive/unstimulated MSCs), in mitigating inflammatory retinal injury.

**Methods:** T-sEV were isolated from TNF-α-preconditioned MSCs and systematically characterized. Small RNA sequencing was performed to identify the microRNA (miRNA) cargo of T-sEV. The effect of T-sEV on lipopolysaccharide (LPS)-induced M1 macrophage polarization was assessed by flow cytometry. Integrated bioinformatic analysis linked T-sEV miRNAs to macrophage transcriptome changes. T-sEV were administered intravitreally in a murine sodium iodate (NaIO_3_)-induced retinal degeneration model. Electroretinography (ERG), optical coherence tomography (OCT), flow cytometry, intraocular pressure (IOP) and systemic examinations were conducted.

**Results:** T-sEV exhibited an enrichment of anti-inflammatory miRNAs, notably miR-146a-5p. They were efficiently internalized by macrophages, significantly suppressing M1 polarization, as evidenced by the decreased percentage of cluster of differentiation (CD)11b^+^/CD86^+^ cells (29.60% ± 2.30%) compared to the sEV group (34.90% ± 1.57%, *P* < 0.05). Analysis showed T-sEV miRNAs targeted and downregulated key pro-inflammatory genes such as Cd86 and Il1r1. *In vivo*, T-sEV treatment significantly preserved retinal a- and b-wave amplitudes and structural integrity. T-sEV treatment markedly reduced retinal macrophage infiltration, decreasing the proportion of F4/80^+^CD11b^+^ cells to 0.38% ± 0.13%, significantly lower than in the sEV group (1.66% ± 0.47%, *P* < 0.01). No adverse effects on IOP or systemic markers were observed.

**Conclusion:** TNF-α preconditioning enhances MSC-derived sEV therapeutic capacity by enriching their anti-inflammatory miRNAs. T-sEV suppress pro-inflammatory macrophage activation and provide superior neuroprotection in a retinal degeneration model, indicating a safe and promising cell-free therapeutic strategy.

## INTRODUCTION

A state of chronic, low-grade inflammation, termed parainflammation, is a key pathogenic driver of neurodegeneration in a broad spectrum of retinal diseases, with atrophic age-related macular degeneration (dry AMD) being a prominent example^[1]^. This persistent inflammatory signaling, often triggered by factors such as complement dysregulation and sustained oxidative stress, not only creates a toxic microenvironment but also directly mediates neuronal injury, culminating in the progressive loss of the retinal pigment epithelium (RPE) and the photoreceptors they support^[2-5]^. A critical therapeutic challenge, therefore, is to simultaneously provide robust neuroprotection and actively counteract this inflammatory microenvironment. To date, no treatments have achieved this dual objective^[6]^.

Mesenchymal stem cells (MSCs; MSC in figures and as an attributive modifier) have been investigated for their paracrine-mediated therapeutic effects^[7-9]^; however, a cell-free approach using MSC-derived small extracellular vesicles (sEV, representing vesicles from naive/unstimulated MSCs) is now favored to circumvent the significant safety and viability concerns associated with direct cell transplantation into the eye^[10,11]^. While sEV from naive MSCs possess baseline therapeutic properties^[12,13]^, their efficacy may be insufficient to overcome the established inflammatory signaling in the degenerating retinal microenvironment. This limitation necessitates strategies to augment the therapeutic potency of sEV.

Preconditioning is an established strategy to bioengineer the cargo of sEV^[14,15]^. It has been proposed that to effectively treat such inflammatory diseases, the therapeutic agent should be primed by an inflammatory signal relevant to the disease’s pathophysiology. Tumor necrosis factor-alpha (TNF-α) is a pleiotropic cytokine known to be upregulated in degenerative retinal diseases^[16-18]^ and is a key mediator of inflammatory pathways, such as the Nuclear factor kappa-light-chain-enhancer of activated B cells (NF-κB) signaling cascade^[19,20]^. Exposing MSCs to TNF-α paradoxically triggers a potent, compensatory anti-inflammatory and pro-survival/neuroprotective program^[21-23]^. This “priming” process modulates the MSCs to release sEV enriched with a specific, therapeutic cargo, such as anti-inflammatory microRNAs, that is functionally tailored to neutralize inflammatory insults^[24]^.

Therefore, we hypothesized that small extracellular vesicles derived from TNF-α-primed MSCs (hereafter abbreviated as T-sEV) represent a targeted therapeutic agent, uniquely equipped to suppress the specific inflammatory pathways active in the degenerating outer retina. Their nano-scale size permits penetration to the RPE and photoreceptor layers following intravitreal (IVT) injection^[25]^. The present study was designed to test this hypothesis by evaluating the efficacy of T-sEV in controlling RPE and photoreceptor degeneration in a preclinical model of oxidative stress-induced retinal inflammatory injury, and to conduct an initial exploration of the molecular mechanisms responsible for their protective effects.

## METHODS

### Animals housing

C57BL/6 mice (Charles River) were housed 4-5 per cage under pathogen-free conditions with ad libitum access to food and water. Environmental controls included a 12 h light-dark cycle, 23 ± 1 °C temperature, and 55% ± 5% humidity. All procedures were approved by the Capital Medical University Laboratory Animal Welfare and Ethical Review (No. AEEI-2022-096). Mice with corneal opacity or other ocular abnormalities, identified by slit-lamp microscopy, were excluded. Humane endpoints included significant weight loss (> 15%) or weakness. For procedures, mice were anesthetized with an intraperitoneal (IP) injection of 0.5% pentobarbital (0.1 mL/10 g·bw). Finally, mice were euthanized by cervical dislocation under deep pentobarbital anesthesia (0.2 mL/10 g·bw).

### Preparation of MSCs

Bone marrow-derived mesenchymal stem cells were isolated from 6-week-old female C57BL/6 mice by whole bone marrow adherence. Marrow was flushed from disinfected femurs and tibias, filtered, and centrifuged (1,500 rpm, 5 min). After red blood cell lysis and two washes, cells were seeded and cultured (37 °C, 5% CO_2_). The medium was first changed after 48-72 h, then every 2-3 days. After 10-14 days, supernatant was collected for sEV enrichment via ultracentrifugation. For sEV collection, cells were cultured in media containing exosome-depleted fetal bovine serum (FBS).

### Preparation of TNF-α-primed MSCs

Based on the above method, MSCs were extracted and stimulated with medium containing mouse TNF-α (10 ng/mL) for 48 h. After washing the cells three times with phosphate buffered saline (PBS), the medium was replaced with fresh medium containing exosome-depleted FBS for continued passaging culture^[21,26,27]^. The culture medium supernatant was collected to enrich T-sEV using ultracentrifugation.

### Extraction of sEV and T-sEV

Cell culture supernatant was centrifuged sequentially at 300 g (10 min) and 2,000 g (10 min) to remove cells. The supernatant was then centrifuged at 10,000 g for 30 min to eliminate cellular debris. Both sEV and T-sEV were pelleted from the resulting supernatant by ultracentrifugation at 100,000 g for 70 min. This pellet was washed by resuspension in sterile PBS followed by a second ultracentrifugation at 100,000 g for 70 min. The final purified vesicles (sEV and T-sEV) pellet was resuspended in sterile PBS. All steps were performed at 4 °C. Purified sEV and T-sEV were immediately aliquoted and stored in PBS at -80 °C for long-term storage, with strict avoidance of repeated freeze-thaw cycles. All sEV and T-sEV utilized in functional experiments were freshly thawed from single-use aliquots.

### TEM imaging acquisition

The sEV and T-sEV were attached to copper grids and stained with uranyl acetate, and the observed images were taken with a JEM-1400 microscope at an acceleration voltage of 120 kV.

### NTA analysis

Control and primed vesicles were diluted to a suitable concentration with 0.22 μm-filtered PBS. The instrument was calibrated with 100 nm standard latex microspheres. Samples were injected into the detection cell via a syringe pump (flow rate 25 μL/s), and particle size, zeta potential and concentration were analyzed at 25 °C.

### Western blot analysis

Appropriate amounts of sEV and T-sEV were mixed with loading buffer and boiled at 95 °C for 10 min, then loaded onto a 4%-20% sodium dodecyl sulfate-polyacrylamide gel electrophoresis (SDS-PAGE) gel. After electrophoresis and membrane transfer, the membrane was blocked for 2 h with skim milk. Primary antibodies for ALIX (ab275377), CD81 (ab109201), TSG101 (#DF8427), and the negative control Histone H3 (YA368) were incubated. Proteins were detected by enhanced chemiluminescence (ECL) after binding to corresponding secondary antibodies. The dilution ratios for primary antibodies were 1:1,000, 1:1,000, 1:1,000, and 1:20,000, respectively.

After enucleation, retinal tissues were rapidly isolated. Total protein was extracted by lysis and quantified. Equal amounts of protein samples were separated by SDS-PAGE and transferred onto poly(vinylidene fluoride) (PVDF) membranes. After blocking, membranes were incubated with primary antibodies against Rhodopsin (ab221664), CD86 (ab220188), Cleaved Caspase-3 (ab214430), and β-actin (YA930, loading control), each at a 1:1,000 dilution. Membranes were then incubated with horseradish peroxidase (HRP)-conjugated secondary antibodies. Protein bands were visualized using an ECL chemiluminescent reagent, imaged, and analyzed.

### Extraction of macrophages

Bone marrow was harvested from the femurs and tibias of 8-week-old female C57BL/6 mice following cervical dislocation. Bones were surface-sterilized with 75% ethanol and cleaned of soft tissue. Marrow was flushed out using cold induction medium, passed through a 70 μm cell filter, and centrifuged (1,500 rpm, 5 min). Red blood cells were subsequently lysed, and the remaining cells were washed with PBS. Finally, cells were cultured for 7 days in bone marrow macrophage induction medium supplemented with macrophage colony-stimulating factor (M-CSF), with the medium replaced every 2-3 days.

### T-sEV and sEV uptake by macrophages or 661W

Purified sEV and T-sEV were fluorescently labeled with Cyanine 5 Succinimidyl Ester (Cy5-SE). Both labeled vesicle types were co-cultured with recipient cells for 10 h, followed by fixation with 4% paraformaldehyde at 37 °C for 15 min. The fixative was removed and cells were washed 2-3 times with PBS. 300 μL of fluorescein isothiocyanate (FITC)-labeled phalloidin (green) was added and incubated at room temperature in the dark for 1 h. The phalloidin was removed and cells washed 2-3 times with PBS. Then 300 μL 4’,6-diamidino-2-phenylindole (DAPI) was added and incubated at room temperature in the dark for 20 min. DAPI was removed and cells washed 2-3 times with PBS. The internalization of labeled sEV and T-sEV by cells was observed using fluorescence microscopy.

### Effect of T-sEV and sEV on the induction of M1 polarization of macrophages

The mature macrophages were digested, replated, and induced to M1 by adding lipopolysaccharide (LPS) (100 ng/mL). At the same time, sEV and T-sEV (200 μg/mL) were added, respectively. PBS alone was added to the control group. The cells were first stained with Zombie Aqua^TM^ Fixable Viability Kit in the dark for 20 min, then washed 2-3 times with PBS (centrifuged at 1,000 rpm for 5 min). Then anti-CD11b-Pacific blue and anti-CD86-Allophycocyanin (APC) were added to the cells for surface staining, 4 °C for 30 min, followed by washing and flow cytometry. Cell culture supernatants were collected, and the concentrations of TNF-α, interleukin-1β (IL-1β), and interferon-γ (IFN-γ) were quantified using Enzyme-linked immunosorbent assay (ELISA) kits according to the manufacturers’ protocols.

### NaIO_3_ administration

The 10-12-week-old male C57BL/6 mice received an IP injection of a 1% (w/v) solution of sodium iodate (NaIO_3_) (S4007, Sigma-Aldrich) in 0.9% saline at a dose of 2.5 μL/g body weight, equivalent to 25 mg/kg, using a 31-gauge, 8-mm-long, 300-μL insulin syringe^[28,29]^.

### Intravitreal injection of T-sEV treatment

Three days after model establishment, treatment was administered via IVT injection. Mice were anesthetized with 0.5% pentobarbital (0.1 mL/10 g·bw), and a Hamilton microinjection syringe was used to inject PBS, sEV, and T-sEV into the vitreous cavity (dosage: The single injection dose per eye was 2 μg of sEV/T-sEV). The dose of 2 μg used in this study was determined based on our laboratory’s previous experience and the physiological volume limits of the mouse eye.

### Imaging of intraocular distribution *in vivo*

Cy5-SE-labeled sEV and T-sEV were intravitreally injected into the eyes of NaIO_3_-treated mice. *In vivo* fluorescence imaging was performed at the indicated time points using the *In Vivo* Imaging System (IVIS) Spectrum system (PerkinElmer), and images were analyzed with Living Image software (version 4.5.5).

### Electroretinography

Retinal function was assessed by full-field electroretinography (ERG). Mice were dark-adapted overnight for 12 h, and all preparations were conducted under dim red light. Mice were anesthetized and their pupils dilated. After corneal anesthesia, the active electrode was placed on the cornea, with the reference electrode subcutaneously on the cheek and a ground electrode at the tail. Scotopic ERG responses were elicited by a 5 ms flash of 3.0 cd·s/m^2^, delivered at 15 s intervals. Stimuli were repeated at least three times for consistency. Waveforms were acquired and analyzed using an Optoprobe Science visual electrophysiology system.

### Optical coherence tomography

Optical coherence tomography (OCT) imaging was performed on day 4 following the IVT injections of PBS, sEV, T-sEV, miR-146a-5p mimic or miR-146a-5p inhibitor. Retinal cross-sectional images of the NaIO_3_-induced model mice were acquired using a commercial ultra-high-resolution ocular imaging system (Optoprobe Science).

### Flow cytometry analysis

For flow cytometry, retinas from enucleated murine eyeballs were processed into single-cell suspensions. The tissue was digested with collagenase IV (1 mg/mL) at 37 °C for 30 min, followed by mechanical dissociation. Cell suspensions were filtered through a 70 μm strainer. Cells were stained with a viability dye, then with anti-F4/80-FITC and anti-CD11b-PB antibodies for 30 min at 4 °C. Data were acquired on a flow cytometer and analyzed using FlowJo.

### Safety assessment

After seven days, intraocular pressure (IOP) was measured using a small animal tonometer. For safety assessment, hematological parameters were analyzed in T-sEV-treated mice and their age-matched normal counterparts.

### miRNA extraction and sequencing

Small RNA sequencing was performed to profile the miRNA cargo of T-sEV and control sEV. Total RNA was extracted from both purified sEV and T-sEV and quality-checked using a NanoDrop ND-1000 and Bioanalyzer 2100. Libraries were constructed via adapter ligation, complementary DNA (cDNA) synthesis, and amplification with Phusion High-Fidelity DNA Polymerase. Libraries were sequenced on an Illumina HiSeq 2500 (50 bp single-end reads). Differentially expressed miRNAs were defined by an absolute log_2_ fold-change (|log_2_FC|) > 1 and an adjusted *P*-value < 0.05.

### mRNA sequencing and analysis

Mature macrophages were M1-polarized with LPS (100 ng/mL), then treated with T-sEV (200 μg/mL) or PBS (control) for 24 h. Total RNA was extracted using TRIzol. Quality-controlled samples (concentration > 50 ng/μL, RIN > 7.0, > 1 μg total RNA) were used for strand-specific library construction. Libraries were prepared via mRNA enrichment, fragmentation, 2’-deoxyuridine 5’-triphosphate (dUTP)-based cDNA synthesis, second-strand digestion, and polymerase chain reaction (PCR) amplification, then sequenced on an Illumina NovaSeq 6000 (PE150). Differentially expressed genes were defined by an absolute |log_2_FC| > 0.586 and a *P*-value < 0.05.

### Bioinformatic analysis of sequencing data

Differentially expressed miRNAs were identified from sequencing data. To handle zero counts in controls, a pseudo-count (1/10,000) was added for correlation analyses, while infinite log_2_FC values were capped for visualization. A high-confidence set of 15 miRNAs was derived by removing non-mouse homologs and retaining those targeting a predefined core gene set (inflammation, oxidative stress, complement mediators).

Target genes for the Sankey diagram were selected via a predefined workflow, prioritizing genes coordinately targeted by multiple miRNAs and those representing central nodes in inflammatory signaling (e.g., Ikbkb, Casp1, Traf1). This process ensured the selection of highly targeted and biologically plausible hubs. Key miRNAs were then prioritized using a non-parametric rank-sum score integrating four criteria: differential expression *P*-value, miRNA log_2_FC, mean log_2_FC of overlapping target genes, and the number of enriched targets.

Enrichment analyses using the Kyoto Encyclopedia of Genes and Genomes/Gene Ontology (KEGG/GO) were performed on the predicted targets of these 15 miRNAs. For validation, miRNA data were integrated with macrophage transcriptomes, defining “overlapping genes” as predicted targets that were also downregulated in T-sEV-treated macrophages. Pathway analysis results for both gene sets were curated to exclude overly broad or irrelevant terms. Complete gene and pathway lists are available in Supplementary Materials.

### Statistical analysis

Statistical analysis was performed using GraphPad Prism 9.5 software. Quantitative data are presented as mean ± standard deviation (SD). Two-tailed unpaired Student’s *t*-test was used for comparison between two groups, and one-way Analysis of Variance (ANOVA) was used for comparison between multiple groups, with Brown-Forsythe or Tukey correction for significance levels. *P* < 0.05 was considered statistically significant. In this study, “*n* = 3” refers to three eyes harvested from three individual mice, representing independent biological replicates (i.e., different individual animals). All analyses were conducted based on these independent samples.

## RESULTS

### Isolation and characterization of T-sEV

**Figure 1:**
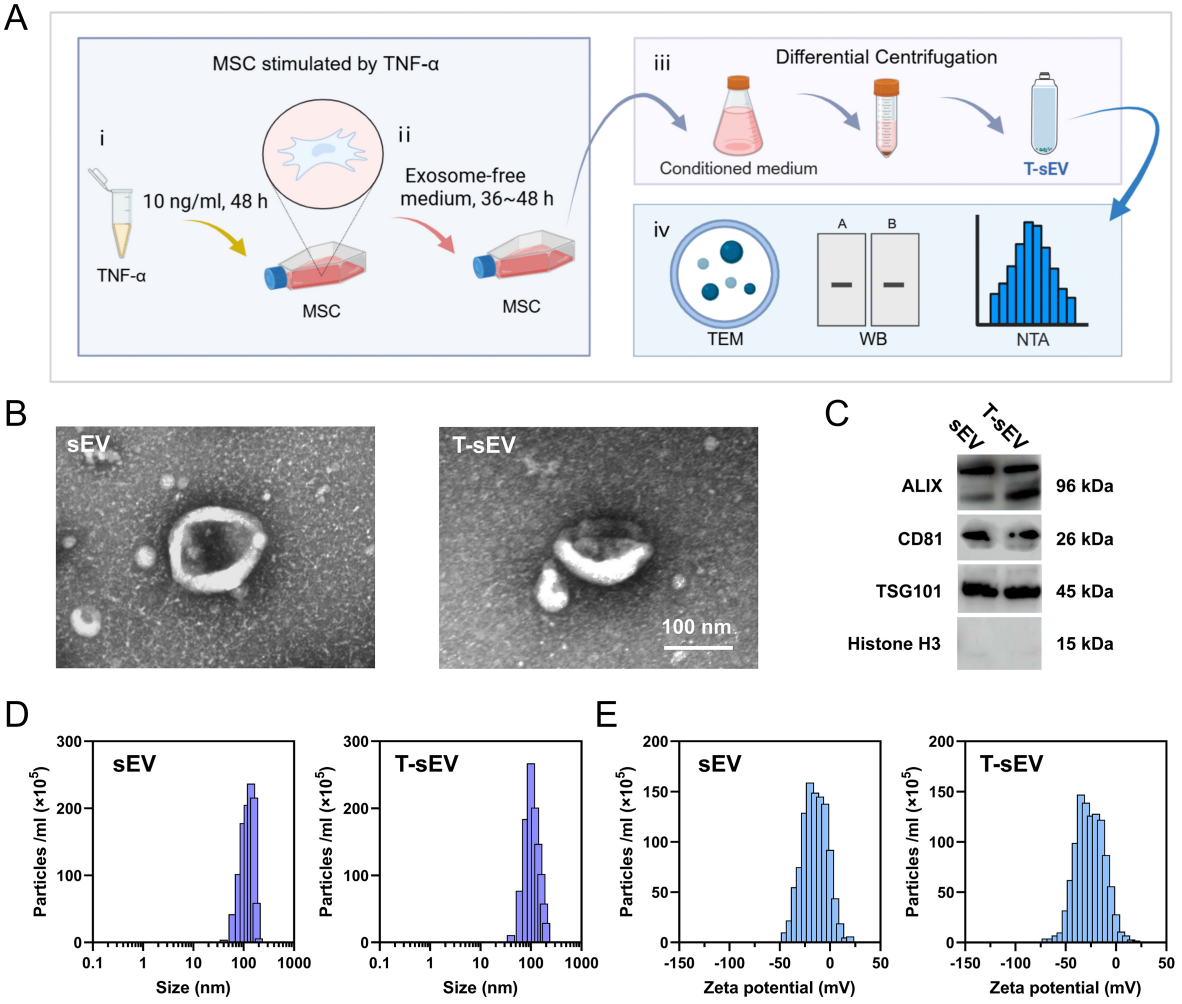
Extraction and characterization of T-sEV. (A) Schematic diagram outlining the key steps for T-sEV generation and analysis: (i) Priming of MSCs with TNF-α (10 ng/mL, 48 h); (ii) Culture in exosome-depleted serum; (iii) Isolation of T-sEV from conditioned medium by ultracentrifugation; (iv) Characterization of isolated T-sEV; (B) Representative TEM image of T-sEV. Scale bar: 100 nm; (C) Western blot analysis of T-sEV lysates probed for the indicated markers (ALIX, CD81, TSG101) and the negative control marker Histone H3; (D and E) NTA of control sEV and T-sEV showed size distribution (D) and characteristic surface charge (E). Figure 1A was created with BioRender.com, and its use complies with BioRender’s licensing agreement. MSC: Mesenchymal stem cell; sEV: small extracellular vesicles, representing vesicles from naive/unstimulated MSCs; T-sEV: sEV derived from TNF-α-primed MSCs; TEM: transmission electron microscopy; WB: western blot; NTA: nanoparticle tracking analysis.

We successfully isolated T-sEV from the conditioned medium of MSCs pretreated with TNF-α, using differential centrifugation. A comprehensive characterization was performed to validate the identity and purity of the isolated vesicles [Figure 1A]. Transmission Electron Microscopy (TEM) analysis revealed that the T-sEV possessed the characteristic cup-shaped or spherical morphology with an intact membrane [Figure 1B]. Concurrently, Western blot analysis verified their vesicular origin by showing robust expression of positive EV markers (ALIX, CD81, and TSG101) and the absence of cellular contamination markers (Histone H3) [Figure 1C]. Furthermore, Nanoparticle Tracking Analysis (NTA) revealed a homogeneous population of isolated particles with a size distribution concentrated below 200 nm and a characteristic zeta potential [Figure 1D and E]. Collectively, these results confirm the efficient isolation of high-purity T-sEV from TNF-α-stimulated MSCs, providing a solid basis for subsequent functional studies.

### Transcriptomic profiling reveals a functionally coordinated anti-inflammatory miRNA cargo in T-sEV

**Figure 2:**
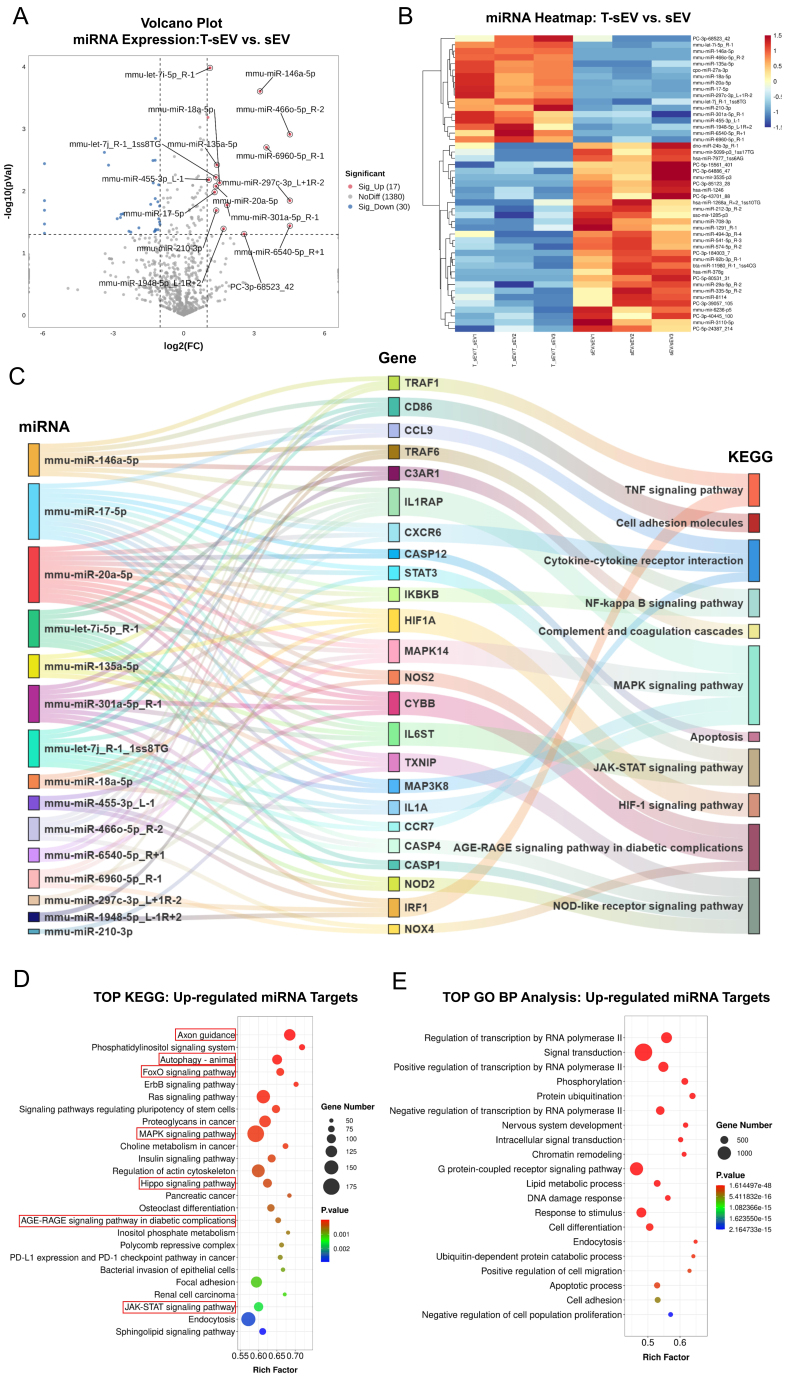
Functional annotation of T-sEV miRNAs through integrated bioinformatic analysis. (A) Volcano plot of differentially expressed miRNAs between T-sEV and sEV. Significantly dysregulated miRNAs were identified using a threshold of |log_2_FC| > 1 and *P*-value < 0.05. Upregulated and downregulated miRNAs are highlighted in red and blue, respectively; (B) Heatmap of significantly dysregulated miRNAs identified in (A), showing hierarchical clustering of miRNAs across samples; (C) Sankey diagram illustrating the network from key upregulated miRNAs in T-sEV (left) to their predicted target genes (center) and subsequently to the significantly enriched KEGG pathways (right); (D) Bubble plot of KEGG pathway enrichment analysis for predicted targets of upregulated T-sEV miRNAs. The top 25 enriched pathways are displayed, selected from the top 30 most significant results (*P*-value < 0.05) by excluding 5 pathways with overly broad functions (see Methods for criteria). The rich factor (X-axis), gene count (bubble size), and statistical significance (color) are indicated; (E) Bubble plot of the top 20 significantly enriched GO biological process terms for the predicted target genes of upregulated T-sEV miRNAs. MSC: Mesenchymal stem cell; sEV: small extracellular vesicles; T-sEV: sEV derived from TNF-α-primed MSCs; FC: fold-change; miRNA: microRNA; KEGG: Kyoto Encyclopedia of Genes and Genomes; GO: Gene Ontology.

The miRNA cargo of T-sEV was significantly altered by TNF-α preconditioning, with small RNA sequencing identifying 17 upregulated miRNAs. After filtering for mouse-specific homologs targeting key pathways, we focused on a high-confidence set of 15 miRNAs to explore their collective function. Among these, the well-established anti-inflammatory regulator mmu-miR-146a-5p showed remarkable enrichment with high statistical significance (adjusted log_2_FC = 3.25; -log_10_P = 3.61). Concurrently, other miRNAs known for broad regulatory roles, such as mmu-miR-17-5p (log_2_FC = 1.32) and mmu-miR-20a-5p (log_2_FC = 1.37), were also consistently upregulated. Notably, a group of miRNAs including mmu-miR-466o-5p_R-2 and mmu-miR-297c-3p_L+1R-2 displayed the highest enrichment levels in the dataset (log_2_FC = 4.52), suggesting they are potent functional components within the vesicles [Figure 2A and B].

To understand the collective biological function of this enriched miRNA signature, we constructed a regulatory network using 15 high-confidence miRNAs. The Sankey diagram revealed that these miRNAs converge to target a core set of genes, which were selected based on being coordinately regulated and their central roles in inflammatory pathways [Figure 2C]. Further functional enrichment analysis of these target genes solidified their biological relevance. KEGG analysis confirmed their significant enrichment in multiple core signaling pathways essential for cellular stress response, survival, and immune regulation, including the mitogen-activated protein kinase (MAPK), forkhead box O (FoxO), and Hippo signaling pathways [Figure 2D]. Complementing this, GO analysis demonstrated that the target genes are strongly involved in fundamental processes such as “nervous system development”, “response to stimulus”, and “apoptotic process” [Figure 2E]. Collectively, these results indicate that the upregulated miRNA population in T-sEV possesses a strong, coordinated potential to modulate cell fate and immune responses.

### T-sEV uptake and immunomodulatory effects on macrophages

**Figure 3:**
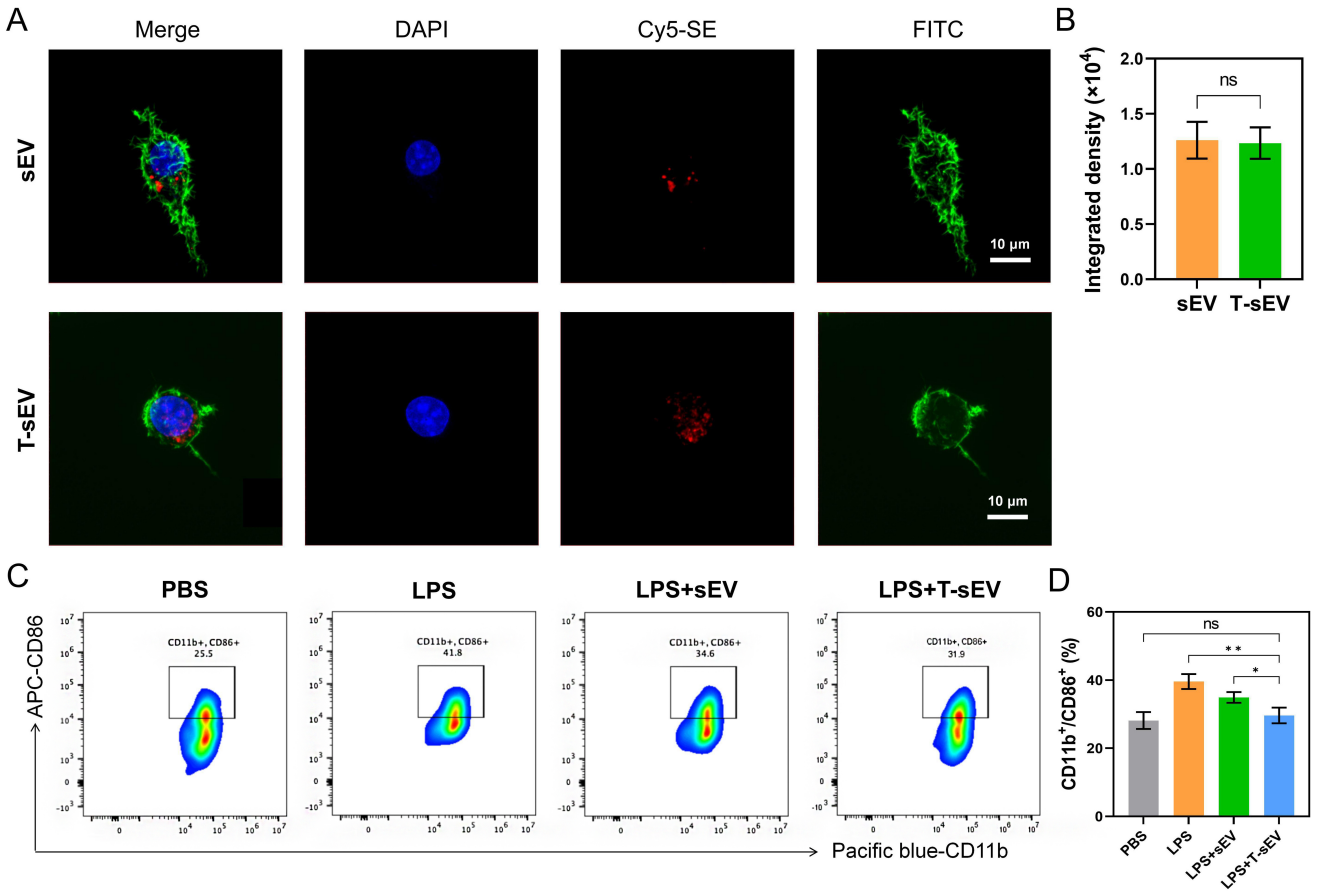
Uptake of T-sEV by macrophages and their effect on macrophage activation. (A) Confocal microscopy images of FITC-labeled cells (green) incubated with Cy5-SE-labeled T-sEV (red). Nuclei are stained with DAPI (blue). Scale bar: 10 μm; (B) Quantification of cellular uptake based on integrated fluorescence density (*n* = 3 biologically independent samples); (C) Representative flow cytometry plots of CD11b^+^/CD86^+^ cells under the indicated treatments (*n* = 3 biologically independent samples); (D) Quantification of CD11b^+^/CD86^+^ macrophages. Data in (B and D) are presented as mean ± SD, and are compared by two-tailed unpaired Student’s *t*-test (B) or one-way ANOVA (D). ^*^P < 0.05; ^**^P < 0.01. MSC: Mesenchymal stem cell; sEV: small extracellular vesicles; T-sEV: sEV derived from TNF-α-primed MSCs; DAPI: 4’,6-diamidino-2-phenylindole; Cy5-SE: cyanine 5 succinimidyl ester; FITC: fluorescein isothiocyanate; CD: cluster of differentiation; SD: standard deviation; ns: no significance.

To assess the function of T-sEV, we first examined their cellular internalization. Both control sEV and T-sEV were labeled with the red fluorescent dye Cy5-SE. Confocal microscopy images showed that both vesicle populations were efficiently internalized by target cells and localized within the cytoplasm [Figure 3A]. Quantitative analysis of the integrated density confirmed no significant difference in uptake efficiency between T-sEV and control sEV, indicating that TNF-α pretreatment did not alter the vesicles’ inherent ability to be taken up by cells [Figure 3B]. Notably, quantitative analysis revealed that the internalization of both T-sEV and sEV was significantly more efficient in macrophages compared to 661W cells (*P* < 0.001), indicating the superior uptake capacity of these immune cells [Supplementary Figure 1]. Subsequently, we evaluated their anti-inflammatory activity in an inflammation model. Compared to the control treatment, the induction of inflammation significantly increased the percentage of activated inflammatory cells, marked by the CD11b^+^/CD86^+^ double-positive population (rising from 28.13% ± 2.47% to 39.57% ± 2.20%). Although control sEV treatment partially inhibited this activation (34.90% ± 1.57%), the T-sEV treatment demonstrated greater efficacy, significantly reducing the CD11b^+^/CD86^+^ population to 29.60% ± 2.30% (*P* < 0.05) [Figure 3C and D]. Furthermore, compared to sEV, T-sEV treatment resulted in significantly greater reductions in the levels of key pro-inflammatory cytokines, including TNF-α (*P* < 0.01), IL-1β (*P* < 0.05), and IFN-γ (*P* < 0.05) [Supplementary Figure 2]. Collectively, these data demonstrate that T-sEV are actively internalized by target cells and exert a powerful inhibitory effect on inflammatory cell activation.

### Integrated analysis reveals T-sEV miRNAs directly reprogram the macrophage inflammatory transcriptome

**Figure 4:**
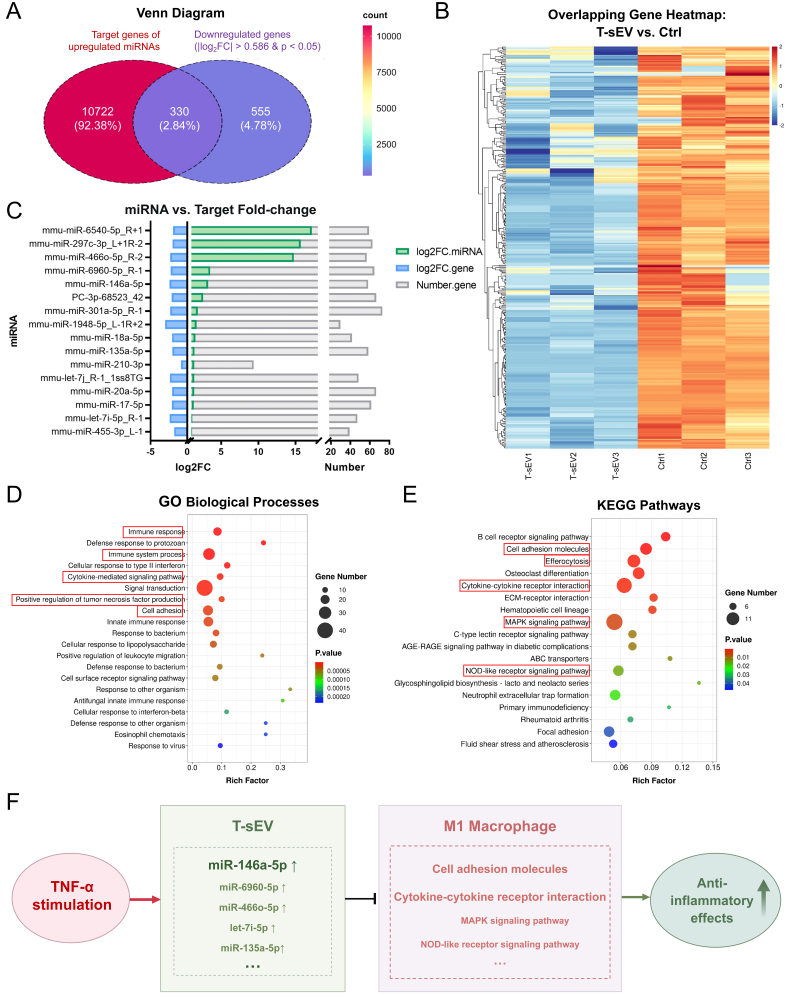
Integrated analysis of T-sEV miRNAs and the transcriptome of recipient macrophages. (A) Venn diagram showing the overlap between the predicted targets of T-sEV-enriched miRNAs (log_2_FC > 1, *P* < 0.05) and the experimentally identified downregulated genes in recipient macrophages (log_2_FC < -0.586, *P* < 0.05). The intersection identifies 330 high-confidence target genes; (B) Heatmap shows the expression patterns of the overlapping genes from (A) across treatment groups; (C) Bar plot showing the inverse expression patterns of selected highly upregulated miRNAs (green bars) and their corresponding, experimentally downregulated target genes (blue bars) derived from the overlapping region in (A). The gray bars represent the count of these downregulated target genes. The x-axis represents the log_2_FC or the gene count; (D) Bubble plot displays the top 20 enriched GO biological process terms for the overlapping genes. Red boxes indicate key terms most relevant to the study’s focus; (E) Bubble plot shows 18 selected KEGG pathways from 33 significantly enriched terms (*P* < 0.05), prioritized based on relevance to immunomodulation (see Methods). Red boxes indicate key pathways most relevant to the study’s focus; (F) Schematic diagram proposes a mechanism by which T-sEV-derived miRNAs regulate macrophage polarization via the key signaling pathways identified above. MSC: Mesenchymal stem cell; sEV: small extracellular vesicles; T-sEV: sEV derived from TNF-α-primed MSCs; FC: fold-change; Ctrl: control; miRNA: microRNA; GO: Gene Ontology; KEGG: Kyoto Encyclopedia of Genes and Genomes.

To establish a direct mechanistic link between the T-sEV miRNA cargo and its immunomodulatory effects, we integrated our sequencing data with the transcriptome profiles of recipient M1 macrophages. This analysis revealed a core set of genes that were both predicted targets of the enriched T-sEV miRNAs and were significantly downregulated in macrophages upon T-sEV treatment [Figure 4A]. Heatmap visualization confirmed the consistent downregulated expression of genes encoding critical pro-inflammatory mediators, such as *Cd86*, *Il1r1*, and *Nos2*, a transcriptional signature that clearly distinguished the T-sEV-treated group from controls [Figure 4B]. Furthermore, this direct regulatory link was further exemplified by the inverse expression patterns observed for specific miRNA-target pairs. For instance, highly enriched miRNAs such as miR-146a-5p and miR-6960-5p showed a strong and corresponding suppression of their respective target genes [Figure 4C]. Gene GO analysis revealed a significant overrepresentation of terms central to immunity, such as “immune response”, “immune system process” and “regulation of tumor necrosis factor production” [Figure 4D]. This was corroborated by KEGG analysis, which showed that the genes clustered in key immunomodulatory pathways, most notably “Cytokine-cytokine receptor interaction” and the “MAPK signaling pathway”, both of which are pivotal in controlling macrophage polarization [Figure 4E].

Based on this multi-layered evidence, we propose a clear mechanistic model: T-sEV deliver a synergistic cargo of miRNAs (with miR-146a-5p as a core component) that collectively suppress critical nodes (including *Cd86*, *Il1r1*, and *Nos2*) within inflammatory signaling networks. This targeted suppression of key pathways, including MAPK signaling, effectively reprograms pro-inflammatory M1 macrophages toward a non-inflammatory, reparative phenotype [Figure 4F]. Together, these findings establish a complete regulatory axis linking the specific miRNA cargo in T-sEV to the functional modulation of macrophage pathways, providing a solid molecular foundation for the therapeutic efficacy observed *in vivo*.

### Enhanced therapeutic efficacy and systemic safety of T-sEV in a murine retinal degeneration model

**Figure 5:**
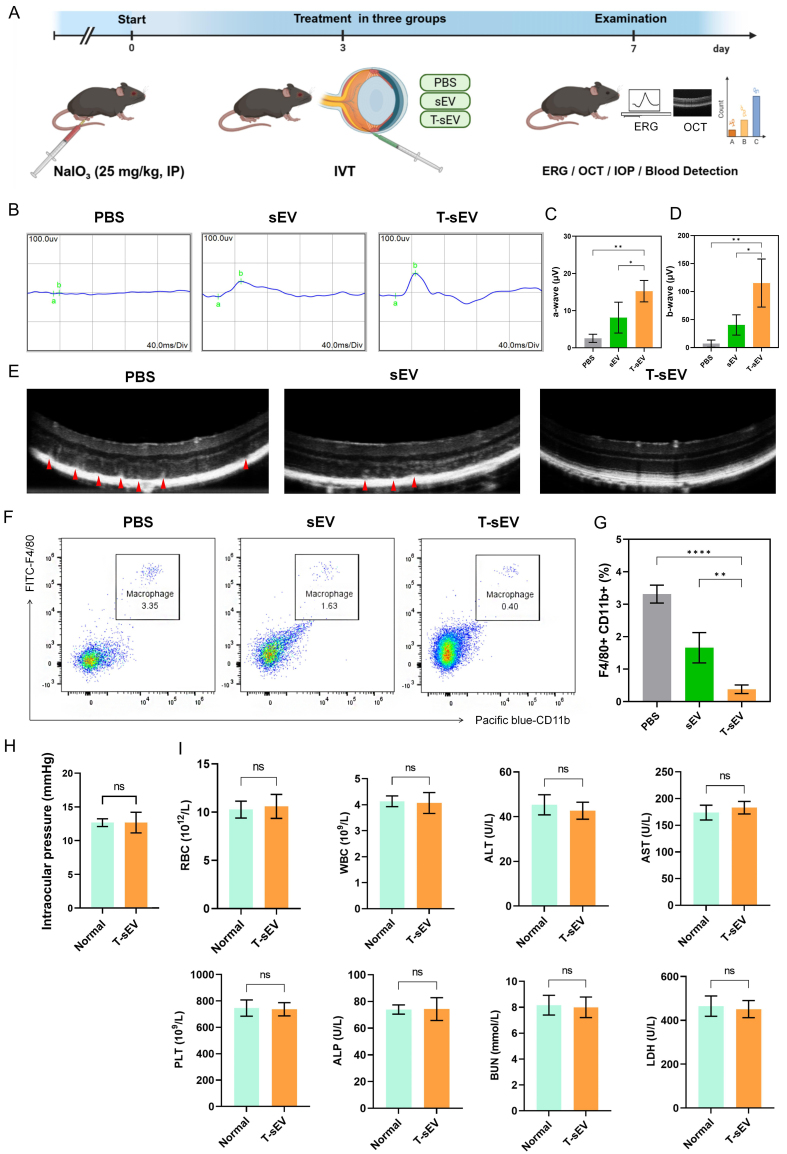
Functional, structural, and systemic safety assessment of T-sEV therapy in a NaIO_3_-induced murine model of retinal degeneration. (A) Experimental timeline outlining key procedures: induction of retinal degeneration, IVT injection of PBS, sEV, or T-sEV on day 3, and final analysis on day 7; (B) Representative electroretinogram waveforms under different treatment conditions; (C and D) Quantitative analysis of a-wave (C) and b-wave (D) amplitudes (*n* = 3, 1 eye from each of 3 mice); (E) Representative OCT images of retinal morphology across treatment groups. Red arrows indicate hyperreflective foci located within the outer retinal layers. Scale bar: 200 μm; (F) Representative flow cytometry plots and (G) quantification of F4/80^+^CD11b^+^ macrophages in retinal tissues (*n* = 3 biologically independent samples); (H) IOP measurements following T-sEV administration (*n* = 3 biologically independent samples); (I) Hematological and serum biochemical parameters after T-sEV treatment (*n* = 3 biologically independent samples). Data in C, D, G, H, I are presented as mean ± SD. Statistical comparisons were performed using one-way ANOVA for panels C, D, and G, and two-tailed unpaired Student’s *t*-test for panels H and I. ^*^P < 0.05; ^**^P < 0.01; ^***^P < 0.001; ^****^P < 0.0001. Figure 5A was created with BioRender.com and its use complies with BioRender’s licensing agreement. NaIO_3_: Sodium iodate; IP: intraperitoneal; MSC: mesenchymal stem cell; sEV: small extracellular vesicles; T-sEV: sEV derived from TNF-α-primed MSCs; PBS: phosphate-buffered saline; IVT: intravitreal; ERG: electroretinography; OCT: optical coherence tomography; IOP: intraocular pressure; CD: cluster of differentiation; RBC: red blood cells; WBC: white blood cells; ALT: alanine aminotransferase; AST: aspartate aminotransferase; PLT: platelets; ALP: alkaline phosphatase; BUN: blood urea nitrogen; LDH: lactate dehydrogenase; SD: standard deviation; ns: no significance.

To validate our *in vitro* findings, we used a murine model of NaIO_3_-induced retinal degeneration that mimics dry AMD. Following NaIO_3_ induction, mice received an IVT of PBS, sEV, or T-sEV on Day 3 and were evaluated on Day 7. Notably, *in vivo* fluorescence imaging demonstrated that T-sEV exhibited prolonged retention in the eye, with fluorescence signals remaining clearly detectable for up to 96 h before significantly diminished by 120 h [Supplementary Figure 3]. Retinal function was assessed by ERG, where the a-wave reflects photoreceptor function, while the b-wave amplitude reflects bipolar and Müller cell function [Figure 5A]. The PBS model group exhibited a near-complete loss of both the photoreceptor-related a-wave and the bipolar-cell-related b-wave signals. Importantly, T-sEV treatment significantly preserved the function of both retinal layers, demonstrating a distinct recovery in a-wave and b-wave amplitudes that was superior to the sEV group [Figure 5B-D]. Structurally, OCT images further confirmed this protective effect, with T-sEV-treated retinas maintaining better structural integrity [Figure 5E]. The potent anti-inflammatory effect of T-sEV was verified by flow cytometry analysis of retinal tissue. The proportion of infiltrating F4/80^+^CD11b^+^ macrophages, which peaked in the PBS group (3.31% ± 0.28%), was significantly suppressed in the T-sEV group (0.38% ± 0.13%), indicating superior inhibition compared to sEV (1.66% ± 0.47%) (*P* < 0.01) [Figure 5F and G]. To further verify the specific contribution of miR-146a-5p *in vivo*, we utilized OCT imaging, which demonstrated that the miR-146a-5p mimic effectively preserved retinal structural integrity compared to the extensive damage observed in the inhibitor group [Supplementary Figure 4A]. Western blot analysis corroborated these findings, showing that the mimic restored Rhodopsin levels while significantly suppressing inflammatory (CD86) and apoptotic (Cleaved Caspase-3) markers [Supplementary Figure 4B].

Finally, T-sEV also exhibited a strong safety profile. Intravitreal administration did not increase IOP [Figure 5H] or alter key hematological and serum biochemical markers [Figure 5I]. These findings confirm the excellent local and systemic biocompatibility of T-sEV.

## DISCUSSION

This study demonstrates that T-sEV have superior therapeutic efficacy compared to naive sEV in a model of inflammatory retinal injury. Our findings indicate that TNF-α priming enriches the sEV cargo with certain specific anti-inflammatory miRNAs. This enhanced payload directly inhibits pro-inflammatory macrophage activation, hence maintaining retinal function and structure.

A key advance of our study is the direct mechanistic linkage from the engineered sEV cargo to the functional therapeutic outcome. We found that TNF-α priming enriched the sEV cargo with functionally relevant anti-inflammatory miRNAs, such as mmu-miR-146a-5p, a well-established negative regulator of the innate immune response and NF-κB signaling^[30-33]^. Our integrated analysis confirmed this mechanism: T-sEV suppressed key pro-inflammatory genes (*Cd86*, *Il1r1*, *Nos2*) in macrophages, inhibiting M1 polarization. This targeted immunomodulation translated to superior *in vivo* efficacy, where T-sEV treatment significantly preserved retinal function (a- and b-waves) and structure (OCT) while markedly reducing macrophage infiltration. This demonstrates a complete pathway from molecular engineering to cellular reprogramming and tissue-level neuroprotection. Furthermore, our analysis identified other highly enriched candidates such as miR-6960-5p and the novel RNA PC-3p-68523_42. Their association with the suppressed inflammatory response suggests T-sEV efficacy is driven by a synergistic miRNA network, positioning them as high-priority targets for future investigation.

The enhanced efficacy of T-sEV is particularly significant when contextualized within the field. While the therapeutic potential of naive sEV is recognized^[34-36]^, our findings underscore their potential limitations in an established inflammatory milieu, where they were consistently outperformed by the preconditioned T-sEV group. Extending previous work on MSC priming^[23,26,37]^, our study elucidates a key therapeutic mechanism of T-sEV in an ocular context. We provide a detailed characterization of the T-sEV miRNA payload and demonstrate its critical role in dictating therapeutic outcomes by modulating macrophage polarization in a model of retinal injury. Inflammatory priming of MSCs with TNF-α, IFN-γ, or IL-1β is a recognized strategy to enhance EV immunomodulatory capacity. Although these stimuli share the goal of boosting potency, they yield distinct cargo profiles. For instance, IFN-γ-primed sEV often enrich indoleamine 2,3-dioxygenase (IDO)^[38,39]^, whereas our study shows that TNF-α specifically enriches miR-146a-5p to modulate macrophage polarization. This specificity underscores our unique contribution and the potential for tailor-made EV therapies through optimized priming for specific disease contexts. Furthermore, the favorable safety profile strongly supports the translational potential of this cell-free therapy.

Several limitations of this study should be acknowledged. Although ideal for assessing immediate immunomodulation, the NaIO_3_ model reflects acute injury rather than the chronic progression of dry AMD^[40]^. Consequently, while our findings demonstrate robust protection at day 7, the long-term durability of a single T-sEV administration remains to be fully elucidated. Future research utilizing chronic degeneration models and extended longitudinal assessments will be essential to determine the optimal dosing frequency and the persistence of the anti-inflammatory response^[41]^. Furthermore, while highlighting miR-146a-5p, we recognize sEV as complex entities where proteins and lipids^[42,43]^ likely work synergistically with miRNAs to mediate the observed effects. Future multi-omics analyses are warranted to elucidate these interactions and comprehensively characterize the T-sEV secretome. Finally, to facilitate clinical translation, future studies should explore a broader range of doses to identify the minimum effective threshold and optimize dosing regimens across different species. Additionally, the limited sample size in the current study necessitates future validation with larger sample sizes to fully address biological variability.

In conclusion, this study demonstrates that TNF-α preconditioning enhances the therapeutic efficacy of MSC-sEV for inflammatory retinal diseases. Through an enriched miRNA cargo, T-sEV suppress pro-inflammatory macrophage activity, leading to significant preservation of retinal structure and function. These findings support engineered sEV as a cell-free therapeutic platform for dry AMD and other retinal neuroinflammatory conditions.

## DECLARATIONS

### Availability of data and materials

The data analyzed during the current study are included in this article’s Supplementary Materials. The raw datasets generated during the study have been deposited in the Open Science Framework repository and are available at: https://osf.io/gb9j7/.

### Copyright

© The Author(s) 2026.
