# Supplementary material for: Extracellular vesicles derived from TNF-α-preconditioned mesenchymal stem cells mitigate inflammatory retinal injury
Source: Extracell Vesicles Circ Nucl Acids. 2026 Mar 17;7(1):377–93. doi: 10.20517/evcna.2025.159 (PMC13074297; doi:10.20517/evcna.2025.159)
Supplement: Supplementary file 1 [file evcna-7-1-377-SupplementaryMaterials.pdf]

Supplementary Materials

Extracellular vesicles derived from TNF-α-preconditioned mesenchymal stem cells mitigate inflammatory retinal injury

Zhuxin Jia[1], Fuxiao Luan[1], Jingyi Shi[1], Yong Tao[1,2,3,4], Ying Tian[1]

[1]Department of Ophthalmology, Beijing Chaoyang Hospital, Capital Medical University, Beijing 100020, China.

[2]National Engineering Research Center for Ophthalmology, Beijing 102600, China.

[3]Engineering Research Center of Ophthalmic Equipment and Materials, Ministry of Education, Beijing 100176, China.

[4]Chinese Institutes for Medical Research, Beijing 100071, China.

Correspondence to: Prof. Ying Tian, Prof. Yong Tao, Department of Ophthalmology, Beijing Chaoyang Hospital, Capital Medical University, Beijing 100020, China. E-mail: tianying@mail.ccmu.edu.cn; taoyong@mail.ccmu.edu.cn

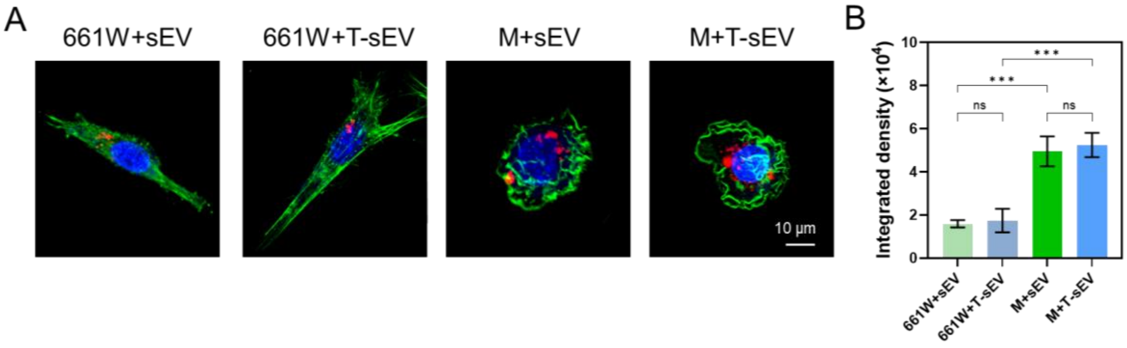

Supplementary Figure 1. Uptake of sEV and T-sEV by 661W cells and macrophages (M). (A) Representative confocal microscopy images of 661W cells and macrophages (stained with FITC, green) incubated with Cy5-SE-labeled sEV or T-sEV (red). Nuclei were stained with DAPI (blue). Scale bar: 10 µm; (B) Quantification of cellular uptake based on the integrated fluorescence density of Cy5 ($n = 3$ biologically independent experiments). Data are presented as mean ± SD and analyzed by one-way ANOVA. ***$P < 0.001$; ns: not significant. The mages were processed using NIS-Elements Viewer 5.21, and graphs were plotted using GraphPad Prism 9.5.

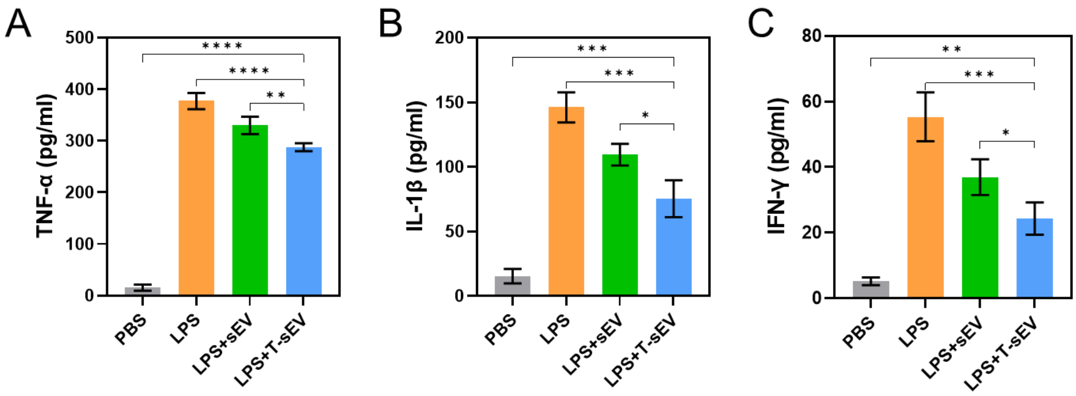

Supplementary Figure 2. Effect of T-sEV on pro-inflammatory cytokine secretion in LPS-stimulated macrophages. Concentrations of TNF-α (A), IL-1β (B), and IFN-γ (C) in culture supernatants were measured by ELISA following the indicated treatments (n = 3 biologically independent experiments). Data are presented as mean ± SD and analyzed by one-way ANOVA. *$P < 0.05$, **$P < 0.01$, ***$P < 0.001$, ****$P < 0.0001$; ns: not significant. The graphs were plotted using GraphPad Prism 9.5.

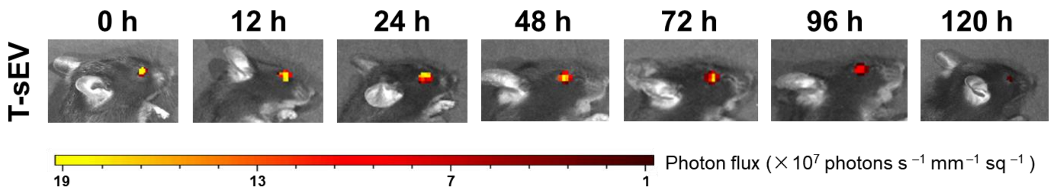

Supplementary Figure 3. *In vivo* evaluation of T-sEV retention in the $NaIO_3$-induced mouse model. Representative in vivo fluorescence images showing the retention of T-sEV in the eye after a single intravitreal injection at the indicated time points. Images were acquired and analyzed using Living Image 4.4 software.

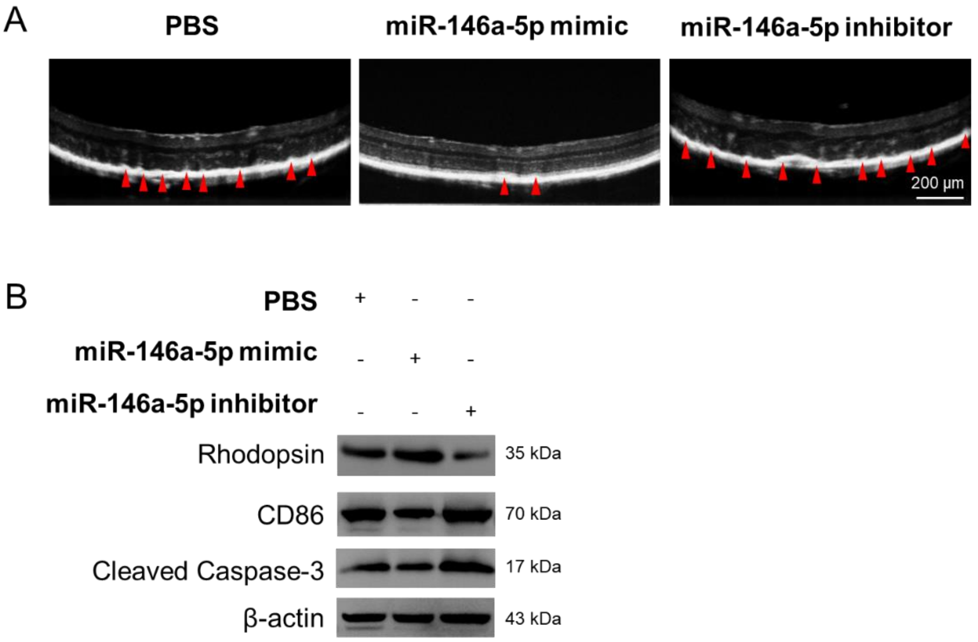

Supplementary Figure 4. Validation of the role of miR-146a-5p *in vivo*. (A) Representative OCT images of retinas from $NaIO_3$-treated mice following intravitreal injection of PBS, miR-146a-5p mimic, or miR-146a-5p inhibitor. Red arrows indicate disruption of the retinal layers (outer nuclear layer/ellipsoid zone). Scale bar: 200 μm; (B) Western blot analysis of Rhodopsin, CD86, and Cleaved Caspase-3 expression in retinal tissues from the indicated groups. β-actin served as a loading control.
